# Azoxystrobin induces mitochondrial dysfunction and mitochondrial pathway apoptosis by targeting the Prx1 Trp87 and Thr90 sites in oral leukoplakia

**DOI:** 10.3389/fphar.2026.1769961

**Published:** 2026-04-20

**Authors:** Wenjing Li, Yajun Shen, Lingyu Li, Min Wang, Min Zhang, Xiaofei Tang

**Affiliations:** 1 Beijing Institute of Dental Research, Beijing Stomatological Hospital and School of Stomatology, Capital Medical University, Beijing, China; 2 Department of Oral and Maxillofacial Surgery, Peking University School and Hospital of Stomatology & National Center for Stomatology & National Clinical Research Center for Oral Diseases & National Engineering Research Center of Oral Biomaterials and Digital Medical Devices & Beijing Key Laboratory of Digital Stomatology & NHC Key Laboratory of Digital Stomatology & NMPA Key Laboratory for Dental Materials, Beijing, China; 3 Department of Oral Pathology, Beijing Stomatological Hospital and School of Stomatology, Capital Medical University, Beijing, China

**Keywords:** azoxystrobin, mitochondrial apoptotic pathway, mitochondrial function, oral leukoplakia, peroxiredoxin 1

## Abstract

**Introduction:**

Oral leukoplakia (OLK) is a prevalent oral potentially malignant disorder with limited treatment options. Our previous research showed that Azoxystrobin (AZOX) induces apoptosis and inhibits mitochondrial complex III activity. Peroxiredoxin 1 (Prx1) plays an important role in OLK progression, and preliminary evidence suggests AZOX may target Prx1 to disrupt mitochondrial function. This study aims to elucidate the precise molecular mechanism by investigating AZOX’s interaction with specific Prx1 residues.

**Methods:**

We employed AutoDock Vina for molecular docking to predict AZOX-Prx1 interactions. Using seamless cloning, Prx1-WT, Prx1-Trp87 mutant, and Prx1-Thr90 mutant variants were constructed and expressed in DOK and Leuk1 OLK cell lines. Comprehensive assessments included cell viability (Cell Counting Kit-8, CCK-8), apoptosis (flow cytometry), mitochondrial ultrastructure (transmission electron microscopy, TEM), mitochondrial ROS (mtROS) production, membrane potential (MMP), complex III activity, cellular energy metabolism (mitochondrial stress assay), and expression of mitochondrial apoptosis-related proteins (Western blot/immunofluorescence).

**Results:**

Molecular docking revealed AZOX forms four hydrogen bonds with the Gln94, Thr90, and Thr49 residues of Prx1 and engages in π-π interaction with Trp87. AZOX treatment significantly inhibited cell proliferation and activated mitochondrial apoptosis, evidenced by increased Bax/Bcl-2 ratio and Cytochrome C (Cyto C) release, which was accompanied by comprehensive mitochondrial dysfunction including structural damage, complex III suppression, elevated mtROS, reduced MMP, and inhibited energy metabolism. Critically, both Trp87 and Thr90 mutations substantially attenuated AZOX’s effects on mitochondrial integrity and apoptotic induction.

**Conclusion:**

AZOX may bind to the Trp87 and Thr90 sites of Prx1 to inhibit mitochondrial function and energy metabolism, and induce mitochondria-mediated apoptosis, thereby suppressing the progression of OLK.

## 1 Introduction

Oral leukoplakia (OLK) is one of the most common oral potential malignant disorders (OPMDs). Studies indicate that the incidence of OLK is approximately 3.4% (Zhang et al., 2023), and more than 9.8% of OLK patients undergo malignant transformation into oral cancer each year (Aguirre-Urizar et al., 2021). Among them, those diagnosed with epithelial dysplasia exhibit a malignant transformation rate of about 34% (Wetzel and Wollenberg, 2020). Currently, drug therapy has become an important direction for OLK treatment. However, existing pharmacological therapies still have limitations, and no treatment regimen with ideal efficacy and broad applicability has been established (Palma et al., 2023). Developing novel, highly effective, and safe therapeutic drugs is of significant clinical importance and research urgency.

Azoxystrobin (AZOX) is a natural fungicide isolated from mushrooms that is employed in the prevention and treatment of fungal diseases (Esser et al., 2004). Its mechanism of action involves the specific inhibition of the ubiquinol oxidation site (Qo) in mitochondrial complex III (Bartlett et al., 2002). We first found that AZOX significantly inhibits the proliferation of dysplastic oral keratinocyte (DOK) cells, CAL27 and SCC15 cells. Oral administration of AZOX markedly suppressed the development of 4-Nitroquinoline N-oxide (4NQO)-induced tongue carcinogenesis in mice by inducing apoptosis. Furthermore, AZOX was found to inhibit mitochondrial function in CAL27 and SCC15 cells (Li et al., 2022a; Chen et al., 2020). Evidence indicates that AZOX suppresses the growth of esophageal cancer cells by inducing mitochondrial pathway apoptosis (Shi et al., 2017). These data suggest that AZOX is a promising candidate drug for preventing OLK carcinogenesis. However, how AZOX exerts its inhibitory effect on OLK progression has not been fully elucidated.

Peroxiredoxin 1 (Prx1), a member of the redox protein family, is widely expressed in living organisms and functions as an antioxidant. It regulates cell growth, differentiation, and death (Neumann et al., 2009). Studies have shown that Prx1 protects mitochondrial function by mitigating lipid peroxidation (Yin et al., 2025). Our previous research found that Prx1 knockdown inhibits 4NQO-induced tongue carcinogenesis in mice. Prx1 silencing induces cellular senescence by mediating the p53/p21 pathway and further promotes mitophagy through its interaction with PHB2/LC3B, thereby suppressing the malignant transformation of OLK (Lu et al., 2021). Additionally, AZOX can dually target the mitochondrial complex III Qo site and Prx1- Cytochrome C1 (CYC1), inhibiting mitochondrial complex III activity, inducing mitochondrial pathway-mediated apoptosis, and consequently suppressing OLK progression (unpublished data). These results indicate that Prx1 may be a potential biological target for AZOX to counteract malignant progression of OLK.

To further elucidate the underlying mechanisms of AZOX functions on OLK, 2 cell lines—DOK and Leuk1 cells with Trp87 and Thr90 mutations in Prx1—were utilized. The impact of these Prx1 site mutations on the regulatory effect of AZOX was investigated by examining mitochondrial function and morphology, cellular energy metabolism levels and mitochondrial apoptosis.

## 2 Materials and methods

### 2.1 Molecular docking

The 3D structure of AZOX (PubChem CID: 3034285) was downloaded from PubChem (https://pubchem.ncbi.nlm.nih.gov/), and the 3D structure of Prx1 (PDB ID: 2RII) was obtained from the PDB database (https://www.rcsb.org/). The Prx1 PDB file was imported into Discovery Studio 2019, processed via the Prepare Protein toolbar to remove non-Prx1 crystal structures, heterocycles and water molecules, and then saved. Molecular docking was conducted using Autodock Vina: the preprocessed Prx1 was imported into AutodockTools as the receptor for dehydration, hydrogenation, charge calculation and atomic type addition. AZOX was imported as the ligand to define its root and select rotatable bonds, with both files saved after preparation. The Prx1 and AZOX structures were opened simultaneously in AutodockTools, the GridBox was set to fully cover Prx1, and docking was launched to generate 10 conformations. The conformation with the highest binding affinity was selected as the optimal one, saved in PDB format and imported into Discovery Studio 2019 for 3D pose adjustment, and the interaction bonds between AZOX and Prx1 were labeled.

### 2.2 Cell culture and Prx1 site-directed mutagenesis in OLK cells

The DOK cell line (ECACC 94122104) was kindly provided by Dr. Qianming Chen of Zhejiang University, China. The DOK cell line is derived from the epithelial dysplasia tissue, providing an effective model for studying the *in vitro* malignant transformation of oral keratinocytes (Chang et al., 1992). The Leuk1 cell line was purchased from Shanghai Xuan Yi Technology Service Center. The Leuk1 cell line is a human oral leukoplakia model derived from a female patient and is widely used in oral precancerous lesion research (Zhu et al., 2021; Guo et al., 2024).

The DOK cells were cultured in high-glucose Dulbecco’s modified Eagle’s medium (Gibco, United States) containing 10% fetal bovine serum (Gibco), 1% penicillin/streptomycin (Biosharp, China), and 5 μg/mL hydrocortisone (Beyotime, China). The Leuk1 cells were cultured in RPMI-1640 medium (Gibco) containing 10% fetal bovine serum (Gibco) and 1% penicillin/streptomycin (Biosharp). Cells were incubated at 37 °C under 5% CO_2_ in a humidified atmosphere.

OLK cells with endogenous Prx1 were used to construct Prx1 mutant cells. Prx1-WT, Prx1-W87A (Trp87 mutated to alanine), and Prx1-T90A (Thr90 mutated to alanine) mutants were constructed using the seamless cloning method (Jiman Biotechnology, China). DOK cells (2 × 10^5^/well) and Leuk1 cells (3 × 10^5^/well) were seeded into 6-well plates. After 24 h of culture, lentivirus was added and incubated for another 24 h. Stably transfected cells were selected with 1 μg/mL puromycin (Sigma, United States) and the transfection efficiency was verified.

### 2.3 Quantitative real-time polymerase chain reaction (qRT-PCR)

Total RNA was isolated from lentivirus-transfected DOK and Leuk1 cells with TRIzol (Thermo Fisher Scientific, United States) and subsequently reverse-transcribed into cDNA using a commercial kit (CoWin Biosciences, China). qRT-PCR was then conducted using SYBR Green PCR Master Mix (CoWin Biosciences) with 1 μL of cDNA. All primers listed in Table 1 were synthesized by Shanghai Jiman Biotechnology.

**TABLE 1:** Sequences of primers used in this study.

Gene	Primer	Sequence
H_Prx1(WT)	Forward	5′-CAT​CTA​GCA​TGG​GTC​AAT​ACA​CCT-3′
​	Reverse	5′-AGG​TCA​TTT​ACA​GTG​ATC​TGC​CG-3′
H_Prx1 (p. W87A)	Forward	5′-CAT​CTA​GCA​GCG​GTC​AAT​ACA​CC-3′
​	Reverse	5′-AGG​TCA​TTT​ACA​GTG​ATC​TGC​CG-3′
H_Prx1 (p. T90A)	Forward	5′-CTA​GCA​TGG​GTC​AAT​GCA​CC-3′
​	Reverse	5′-AGG​TCA​TTT​ACA​GTG​ATC​TGC​CG-3′
GAPDH	Forward	5′-TCT​CCA​CAC​CTA​TGG​TGC​AA-3′
​	Reverse	5′-CAA​GAA​ACA​GGG​GAG​CTG​AG-3′

### 2.4 Cell viability assay

Building on our previous finding that 10 μg/mL AZOX inhibits DOK cell proliferation and induces apoptosis (Li et al., 2022a), we treated DOK and Leuk1 cells transfected with wild-type Prx1, Trp87-mutated Prx1, or Thr90-mutated Prx1 with 0 or 10 μg/mL of AZOX for 12, 24, and 48 h. Cell proliferation was then assessed via Cell Counting Kit-8 (CCK-8; Dojindo, China) by measuring the absorbance at 450 nm after a 2-h incubation with CCK-8 working solution (10 μL CCK-8:100 μL medium).

### 2.5 Apoptosis analysis

After being treated with AZOX at 0 or 10 μg/mL for 24 and 48 h, DOK and Leuk1 cells transfected with wild-type Prx1, Trp87-mutated Prx1, or Thr90-mutated Prx1 were incubated with Annexin V-fluorescein isothiocyanate and PI for 15 min in the dark. Apoptotic rates were analyzed by flow cytometry (BD, United States).

### 2.6 Transmission electron microscopy (TEM)

After treatment with 0 or 10 μg/mL AZOX for 24 h, DOK cells and Leuk1 cells transfected with wild-type Prx1, Trp87-mutated Prx1, or Thr90-mutated Prx1 were collected. To evaluate the cellular ultrastructure, cells were fixed with 2.5% glutaraldehyde and processed for TEM. The samples were subsequently dehydrated through a graded series of ethanol and propylene oxide, thin-sectioned, and stained with 0.3% lead citrate. Ultrastructural examination of organelles, including mitochondria, the endoplasmic reticulum, and lipid droplets, was performed (JEM-2100, Japan).

### 2.7 Measurement of intracellular mitochondrial ROS (mtROS)

Following a 24-h treatment with 0 or 10 μg/mL AZOX, DOK and Leuk1 cells, which had been transfected with wild-type Prx1, Trp87-mutated Prx1, or Thr90-mutated Prx1 respectively, were collected. mSoxUp was applied for 4 h, serving as the positive control. DOK cells and Leuk1 cells were stained in the dark for 30 min at 37 °C by Mito-SOX Red (Beyotime). Fluorescence intensity was detected using flow cytometry (Violet 610). Cells in 6-well plates were subjected to staining with fluorescent probe staining solution (MitoSOX Green, Thermo, United States) by incubation at 37 °C for 15 min in darkness. Subsequent fluorescence imaging was observed on an Olympus BX61 microscope configured for 488 nm excitation and 510 nm emission.

### 2.8 Detection of mitochondrial complex III activity

Subsequent to a 24-h exposure to 0 or 10 μg/mL AZOX, DOK and Leuk1 cells were collected, which had been transfected with wild-type Prx1, Trp87-mutated Prx1, or Thr90-mutated Prx1, respectively. The activity of mitochondrial complex III was measured using a commercial assay kit (Solarbio, China), and the protein concentration was determined via a standard curve. The specific activity (U/mg prot) was calculated according to the manufacturer’s guidelines.

### 2.9 Measurement of mitochondrial membrane potential (MMP)

After completing a 24-h treatment with 0 or 10 μg/mL AZOX, DOK and Leuk1 cells, which had been transfected with wild-type, Trp87-mutant, or Thr90-mutant Prx1, were incubated with JC-1 staining solution at 37 °C for 20 min. Carbonyl cyanide-chlorophenylhydrazone (CCCP) was applied for 30 min, serving as the positive control. JC-1 monomers were detected at 490 nm excitation and 530 nm emission (green fluorescence), while JC-1 aggregates were measured at 525 nm excitation and 590 nm emission (red fluorescence). Mitochondrial membrane potential (MMP) was quantified as the ratio of red to green fluorescence intensity using flow cytometry. Images were acquired using an Olympus BX61 microscope.

### 2.10 Cell mitochondrial stress assay

The oxygen consumption rate (OCR) was measured with a Seahorse XFe24 Real-Time Cell Metabolism Analyzer (Agilent, United States) using the Seahorse XF Cell Mito Stress Test Kit. Leuk1 cells were plated in 24-well Seahorse microplates at 6.8 × 10^3^ cells/well, were allowed to adhere overnight and then treated with 0 or 10 μg/mL AZOX for 24 h. Following treatment, the cells were washed with Seahorse XF assay medium. The OCR was then measured by automated injection of oligomycin (1.5 µM), carbonyl cyanide 4-(trifluoromethoxy) phenylhydrazone (FCCP) (0.5 µM), and a rotenone/antimycin A mixture (0.5 µM each). OCR values were normalized to the number of cells in each plate determined at the time of measurement.

### 2.11 Live cell imaging

DOK and Leuk1 cells were seeded into confocal dishes and cultured routinely for 24 h. Mitochondrial staining was performed using the Mito-Tracker Green (Beyotime) at 37 °C, 60 nM for 23 min for DOK cells and 50 nM for 15 min for Leuk1 cells. Nuclear staining was then carried out using Hoechst 33342 (Solarbio) at 1 μg/mL for 20 min at 37 °C. After staining, 10 μg/mL Cy5-labeled AZOX was added, and fluorescence signals were immediately observed and captured under a Keyence BZ-X800LE microscope.

### 2.12 Western blotting

After a 24-h exposure to 0 or 10 μg/mL AZOX, DOK and Leuk1 cells were harvested. Total proteins were extracted from cells following standard protocol. Protein concentration was measured using the Bradford method. Equal amounts of protein samples were separated on a 10% gel via sodium dodecyl sulfate-polyacrylamide gel electrophoresis, transferred to a polyvinylidene difluoride membrane, and blocked with 5% skim milk for 1 h at room temperature. After incubation with primary antibodies against Bax (1:1,000, Abcam), Bcl-2 (1:1,000, Abcam), Cytochrome C (Cyto C) (1:5,000, Proteintech) and VCL (1:1,000, CST) overnight at 4 °C, the membrane was incubated with the appropriate secondary antibody for 1 h at room temperature and detected using an enhanced chemiluminescence reagent (BioRad, United States).

### 2.13 Immunofluorescence assay

Upon completing a 24-h treatment with 0 or 10 μg/mL AZOX, DOK and Leuk1 cells were fixed with 100% ethanol for 20 min, washed with phosphate-buffered saline (PBS), permeabilized with 0.5% Triton for 15 min, and blocked with 1% bovine serum albumin for 1 h. The cells were incubated with primary antibodies against Bax (1:400, Proteintech), Bcl-2 (1:50, Abclonal), Cyto C (1:200, Proteintech), Caspase3 (1:100, Proteintech) and Caspase9 (1:200, Proteintech) overnight at 4 °C. The cells were rinsed three times with PBS and incubated with the fluorescent secondary antibody (1:100, Abclonal) for 1 h. To visualize the nuclei, cells were incubated with 4′,6-Diamidino-2′-phenylindole (DAPI) (Beyotime, China) for 5 min, and the fluorescence signal was measured using the fluorescence microscope (IX71; OLYMPUS).

### 2.14 Statistical analysis

Statistical analysis was performed using IBM SPSS v.20.0. Data, expressed as mean ± SD, underwent tests for normality and homogeneity of variance. For comparisons between two groups, Student’s t-test was employed. For multiple group comparisons, one-way analysis of variance (ANOVA) was conducted, followed by Bonferroni *post hoc* test to correct for multiple comparisons. *P* < 0.05 was considered statistically significant.

## 3 Result

### 3.1 Prediction of the interaction sites of AZOX with Prx1

**FIGURE 1:**
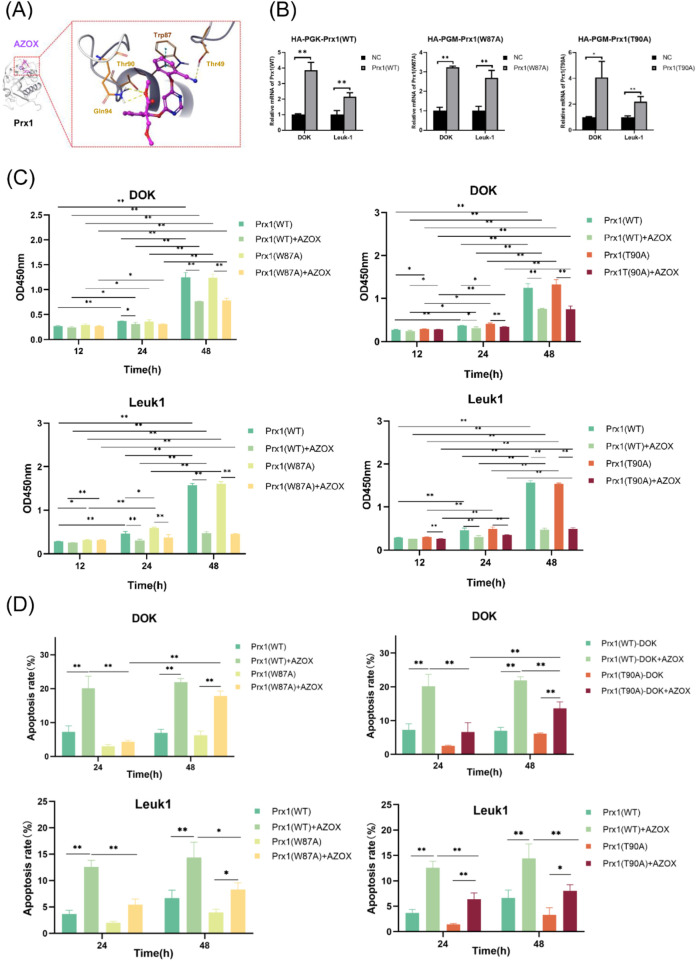
Prx1 mutations diminish the pro-apoptotic and anti-proliferative effects of AZOX on OLK cells. **(A)** 3D docking conformation of AZOX with Prx1. **(B)**Validation of transfection efficiency of wild-type Prx1, Trp87-mutated Prx1, and Thr90-mutated Prx1 in OLK cells. **(C)** CCK-8 assay detected the effect of AZOX on cell proliferation in OLK cells. **(D)** Flow cytometry detected apoptosis levels in OLK cells after AZOX treatment (*P < 0.05; **P < 0.01; n = 3).

The prediction of the interaction sites of AZOX with Prx1 was conducted by AutoDock Vina. Using Autodock Vina software, we simulated whether AZOX directly interacts with Prx1. The conformation with the minimum binding energy was considered the optimal binding position. The results showed that AZOX bound directly to Prx1, with the strongest binding affinity of −8.1 kcal/mol observed at the optimal binding conformation. Specifically, AZOX formed four hydrogen bonds with the Gln94, Thr90, and Thr49 residues of Prx1 protein, and a π-π stacking interaction at the Trp87 residue (Figure 1A). These findings suggest that AZOX may directly target Prx1, thereby exerting its effect on OLK.

Previous studies have shown that phosphorylation of Thr90 in Prx1 inactivates its peroxidase activity and enhances its chaperone activity (Lim et al., 2015; Chang et al., 2002; Jang et al., 2006). Given that AZOX can form a specific π-π interaction with Trp87 of Prx1, both Trp87 and Thr90 were selected as the target sites for subsequent Prx1 mutations in this study.

### 3.2 The mutations of Trp87 and Thr90 in Prx1 were established successfully in DOK and Leuk1 cells

The wild-type Prx1, Trp87-mutated Prx1, and Thr90-mutated Prx1 were transfected into DOK and Leuk1 cells. The successful establishment of the DOK and Leuk1 cell lines, which expressed wild-type Prx1, Trp87-mutated Prx1, or Thr90-mutated Prx1 respectively, was verified by qRT-PCR (Figure 1B).

### 3.3 Prx1 mutations had no significant effect on the anti-proliferative action of AZOX against OLK cells

To investigate the effects of AZOX on cell viability in OLK cells and the impact of Trp87 and Thr90 mutations in Prx1 on the regulatory effect of AZOX, CCK-8 assay was conducted. The results showed that AZOX treatment significantly inhibited the proliferation of both DOK (Table 2, rows 1-2) and Leuk1 (Table 2, rows 3-4) cells after 24 h and 48 h in a time-dependent manner (Figure 1C). At 12 h post-AZOX treatment, cell proliferation was slightly higher in Trp87-mutated and Thr90-mutated Prx1-transfected OLK cells compared to wild-type Prx1-transfected OLK cells (Table 2, rows 5-6); however, AZOX did not significantly suppress OLK cell viability at this time point (Figure 1C).

**TABLE 2:** Quantitative data corresponding to Result 3.3.

Row	Cell lines	Groups	Mean ± SD	Change (%)
1	DOK	24 h-Prx1-WT	0.37 ± 0	↓16%
​	​	24 h-Prx1-WT + AZOX	0.31 ± 0.03	
2	DOK	48 h-Prx1-WT	1.25 ± 0.1	↓39%
​	​	48 h-Prx1-WT + AZOX	0.76 ± 0.01	
3	Leuk1	24 h-Prx1-WT	0.46 ± 0.05	↓33%
​	​	24 h-Prx1-WT + AZOX	0.31 ± 0.03	
4	Leuk1	48 h-Prx1-WT	1.57 ± 0.04	↓70%
​	​	48 h-Prx1-WT + AZOX	0.47 ± 0.04	
5	DOK	12 h-Prx1-WT + AZOX	0.24 ± 0.02	↑17%
​	​	12 h- Prx1-T90A + AZOX	0.28 ± 0	
6	Leuk1	12 h-Prx1-WT + AZOX	0.27 ± 0.01	↑19%
​	​	12 h- Prx1-W87A + AZOX	0.32 ± 0.01	

### 3.4 Prx1 mutations diminish AZOX-induced pro-apoptosis in OLK cells

To investigate whether AZOX inhibits proliferation by inducing apoptosis, flow cytometry was performed. The results showed that AZOX treatment for 24 and 48 h significantly induced apoptosis in both DOK and Leuk1 cells (Table 3, rows 1–4; Figure 1D). Despite receiving the same AZOX treatment, OLK cells carrying Prx1 mutations showed reduced apoptosis rate relative to wild-type Prx1-transfected cells (Table 3, rows 5–8; Figure 1D). Therefore, we speculate that the Trp87 and Thr90 sites of Prx1 may mediate the pharmacological effects of AZOX by inducing apoptosis in OLK cells.

**TABLE 3:** Quantitative data corresponding to Result 3.4.

Row	Cell lines	Groups	Mean ± SD	Change (%)
1	DOK	24 h-Prx1-WT	7.29 ± 1.76	↑176%
​	​	24 h-Prx1-WT + AZOX	20.13 ± 3.58	
2	DOK	48 h-Prx1-WT	6.97 ± 1.04	↑214%
​	​	48 h-Prx1-WT + AZOX	21.92 ± 1.06	
3	Leuk1	24 h-Prx1-WT	3.7 ± 0.69	↑241%
​	​	24 h-Prx1-WT + AZOX	12.6 ± 1.27	
4	Leuk1	48 h-Prx1-WT	6.68 ± 1.51	↑116%
​	​	48 h-Prx1-WT + AZOX	14.41 ± 2.86	
5	DOK	24 h-Prx1-WT + AZOX	20.13 ± 3.58	↓78%
​	​	24 h-Prx1-W87A + AZOX	4.37 ± 0.41	
6	DOK	24 h-Prx1-WT + AZOX	20.13 ± 3.58	↓67%
​	​	24 h-Prx1-T90A + AZOX	6.64 ± 2.78	
7	Leuk1	24 h-Prx1-WT + AZOX	12.6 ± 1.27	↓57%
​	​	24 h-Prx1-W87A + AZOX	5.43 ± 1.07	
8	Leuk1	24 h-Prx1-WT + AZOX	12.6 ± 1.27	↓49%
​	​	24 h-Prx1-T90A + AZOX	6.43 ± 1.20	

### 3.5 Prx1 mutations alleviate AZOX-induced organelle damage in OLK cells

**FIGURE 2:**
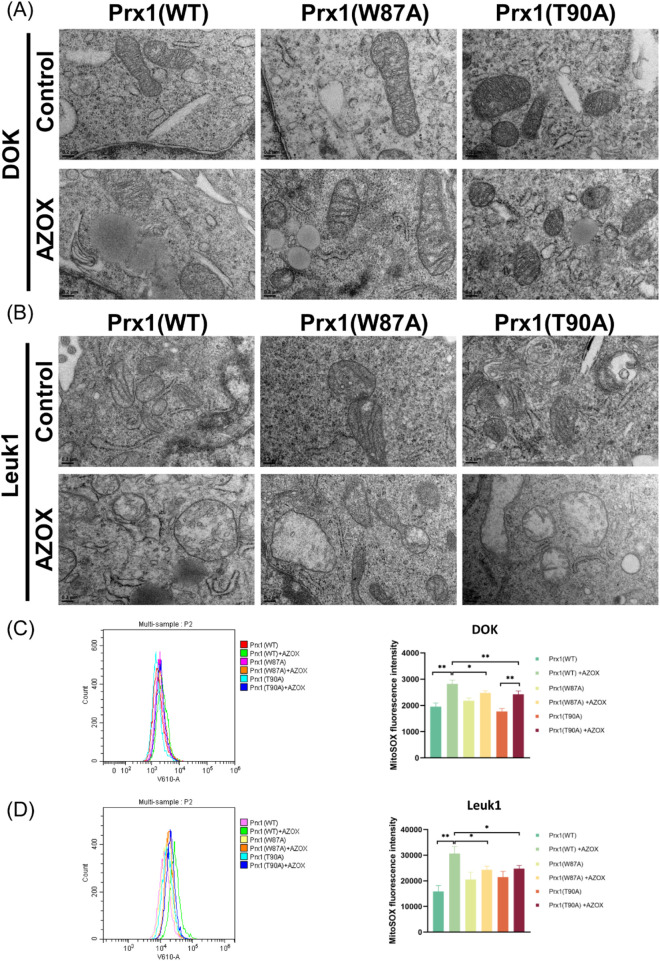
AZOX induces ultrastructural changes and mtROS upregulation in OLK cells, while Prx1 mutations reduce its efficacy **(A)** Micrographs were taken under a TEM showing mitochondria, endoplasmic reticulum, and lipid droplets in DOK cells (magnification 8,000×). **(B)** Micrographs were taken under a transmission electron microscope showing mitochondria, endoplasmic reticulum, and lipid droplets in Leuk1 cells (magnification 8,000×). **(C)** Detection of mtROS levels in DOK cells following AZOX treatment and Prx1 mutations using flow cytometry. **(D)** Detection of mtROS levels in Leuk1 cells following AZOX treatment and Prx1 mutations using flow cytometry (*P < 0.05; **P < 0.01; n = 3).

To investigate the effects of AZOX on mitochondrial function in OLK cells and the impact of Trp87 and Thr90 mutations in Prx1 on its regulatory effect, TEM was employed to examine the ultrastructural changes in OLK cells. The results revealed that after AZOX treatment, DOK cells exhibited mitochondrial swelling, disruption of the mitochondrial double-membrane structure, discontinuous mitochondrial cristae, extensive endoplasmic reticulum dilation, and increased lipid droplets (Figure 2A). Similar morphological changes were also observed in AZOX-treated DOK cells transfected with Prx1 Trp87 or Thr90 mutations. In Leuk1 cells transfected with wild-type Prx1, mitochondrial morphology remained normal; however, slight mitochondrial swelling was observed in Leuk1 cells carrying Trp87 or Thr90 mutations. Following AZOX treatment, all Leuk1 cells transfected exhibited extensive mitochondrial swelling, disruption of the double-membrane structure, discontinuous mitochondrial cristae, significant dilation of the endoplasmic reticulum, and an increase in lipid droplet accumulation (Figure 2B). These findings suggest that AZOX damages OLK cellular ultrastructure and the Thr90 and Trp87 residues of Prx1 play a critical role in regulating mitochondrial function and maintaining mitochondrial homeostasis.

### 3.6 Prx1 mutations decrease AZOX-induced mtROS levels in OLK cells

**FIGURE 3:**
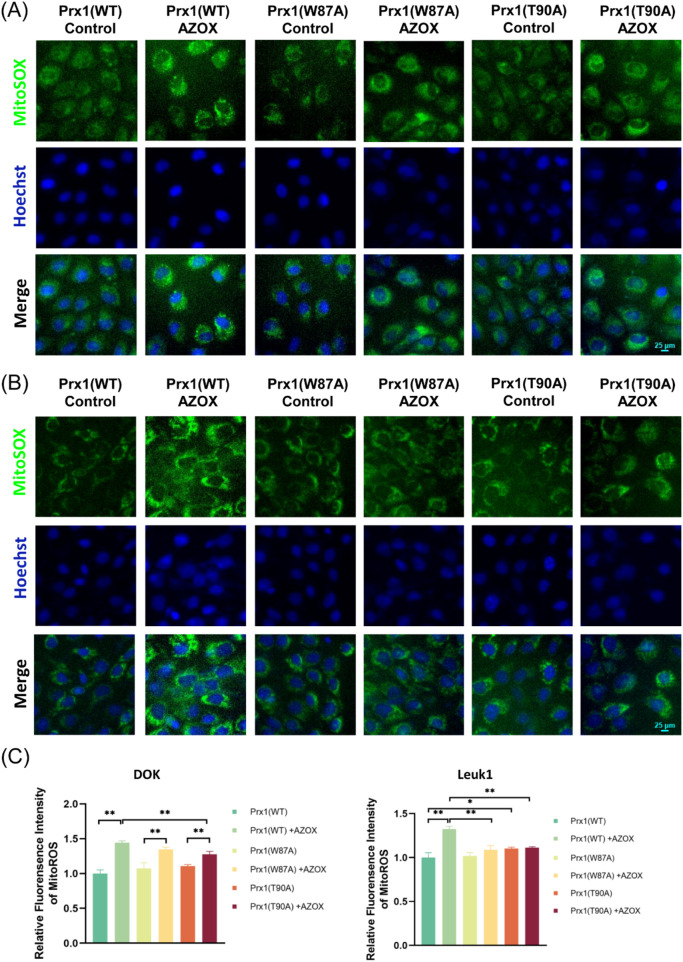
AZOX induces oxidative stress in OLK cells, which is attenuated after Prx1 mutation. **(A)** The effect of AZOX treatment and Prx1 mutation on mtROS levels in DOK cells was observed under a fluorescence microscope (magnification ×200). **(B)** The effect of AZOX treatment and Prx1 mutation on mtROS levels in Leuk1 cells was observed under a fluorescence microscope (magnification ×200). **(C)** Statistical graph of mtROS levels in DOK cells and Leuk1 cells (*P < 0.05; **P < 0.01; n = 3).

mtROS is a major contributor to mitochondrial dysfunction. MitoSox staining is commonly used to detect the production of mtROS. As shown in Figure 3, AZOX induced an increase in mtROS levels in OLK cells (Table 4, rows 1-2), thereby inducing oxidative stress. Compared to OLK cells transfected with wild-type Prx1, the mutation in Thr90 of Prx1 resulted in increased mtROS levels (Table 4, row 3). Following AZOX treatment, OLK cells with Prx1 mutations also underwent oxidative stress; however, compared to OLK cells transfected with wild-type Prx1, the increase in mtROS were less pronounced (Table 4, rows 4–6). Results from flow cytometry were consistent with the aforementioned trend (Figures 2C,D).

**TABLE 4:** Quantitative data corresponding to Result 3.6.

Row	Cell lines	Groups	Mean ± SD	Change (%)
1	DOK	Prx1-WT	1 ± 0.05	↑44%
​	​	Prx1-WT + AZOX	1.44 ± 0.03	
2	Leuk1	Prx1-WT	1 ± 0.06	↑32%
​	​	Prx1-WT + AZOX	1.32 ± 0.03	
3	Leuk1	Prx1-WT	1 ± 0.06	↑10%
​	​	Prx1-T90A	1.1 ± 0.01	
4	DOK	Prx1-WT + AZOX	1.44 ± 0.03	↓11%
​	​	Prx1-T90A + AZOX	1.28 ± 0.04	
5	Leuk1	Prx1-WT + AZOX	1.32 ± 0.03	↓18%
​	​	Prx1-W87A + AZOX	1.08 ± 0.05	
6	Leuk1	Prx1-WT + AZOX	1.32 ± 0.03	↓16%
​	​	Prx1-T90A + AZOX	1.11 ± 0.01	

### 3.7 AZOX inhibits mitochondrial complex III activity in OLK cells attenuated by Prx1 mutations

**FIGURE 4:**
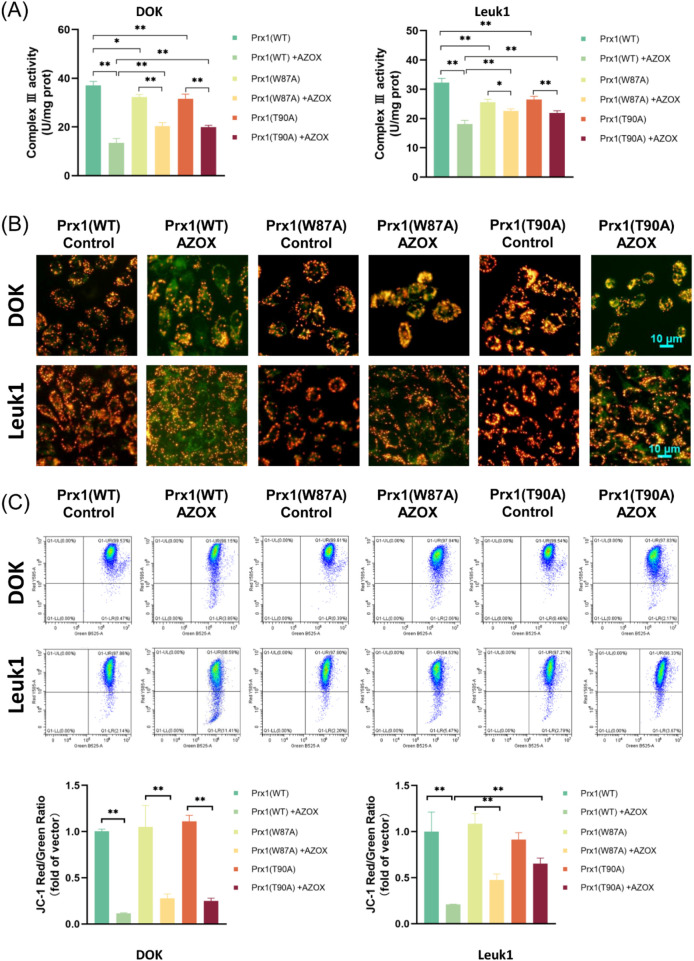
AZOX-induced mitochondrial dysfunction in OLK cells attenuated by Prx1 mutations, **(A)** Changes in mitochondrial complex III activity in OLK cells following AZOX treatment and Prx1 mutations, **(B)** The effect of AZOX treatment and Prx1 mutations on the MMP of OLK cells were observed under a microscope (magnification ×200), **(C)** Flow cytometric detected the effects of AZOX treatment and Prx1 mutations on MMP in OLK cells (*P < 0.05; **P < 0.01; n = 3).

Mitochondrial complex III activity was measured using a commercial assay kit. The results demonstrated that 24-h AZOX treatment significantly inhibited mitochondrial complex III activity in OLK cells (Table 5, rows 1-2). A decrease in mitochondrial complex III activity was observed following mutations at Trp87 and Thr90 of Prx1, relative to the wild-type control in OLK cells (Table 5, rows 3–6). However, in Prx1-mutated OLK cells, the AZOX-induced reduction in complex III activity was attenuated compared to wild-type Prx1-transfected cells, indicating a decrease in AZOX efficacy (Table 5, rows 7–10; Figure 4A).

**TABLE 5:** Quantitative data corresponding to Result 3.7.

Row	Cell lines	Groups	Mean ± SD	Change (%)
1	DOK	Prx1-WT	37.1 ± 1.64	↓64%
​	​	Prx1-WT + AZOX	13.49 ± 1.83	
2	Leuk1	Prx1-WT	32.23 ± 1.52	↓44%
​	​	Prx1-WT + AZOX	18.09 ± 1.32	
3	DOK	Prx1-WT	37.1 ± 1.64	↓13%
​	​	Prx1-W87A	32.29 ± 1.07	
4	DOK	Prx1-WT	37.1 ± 1.64	↓15%
​	​	Prx1-T90A	31.6 ± 1.91	
5	Leuk1	Prx1-WT	32.23 ± 1.52	↓21%
​	​	Prx1-W87A	25.58 ± 0.98	
6	Leuk1	Prx1-WT	32.23 ± 1.52	↓18%
​	​	Prx1-T90A	26.53 ± 1.02	
7	DOK	Prx1-WT + AZOX	13.49 ± 1.83	↑51%
​	​	Prx1-W87A + AZOX	20.36 ± 1.49	
8	DOK	Prx1-WT + AZOX	13.49 ± 1.83	↑48%
​	​	Prx1-T90A + AZOX	19.92 ± 0.79	
9	Leuk1	Prx1-WT + AZOX	18.09 ± 1.32	↑25%
​	​	Prx1-W87A + AZOX	22.6 ± 0.71	
10	Leuk1	Prx1-WT + AZOX	18.09 ± 1.32	↑21%
​	​	Prx1-T90A + AZOX	21.95 ± 0.7	

### 3.8 Prx1 mutations affect MMP levels induced by AZOX in OLK cells

Disruption of MMP is a key indicator of mitochondrial dysfunction. After 24 h of AZOX treatment, OLK cells were collected and stained with the JC-1 fluorescent probe. Flow cytometry and microscopic observation revealed that, compared with the control group, AZOX-treated groups showed enhanced green fluorescence and decreased MMP, indicating early-stage apoptosis (Table 6, rows 1-2). However, the enhancement of green fluorescence was less pronounced in Prx1-mutated OLK cells than in wild-type Prx1-transfected cells, suggesting an attenuated effect of AZOX (Table 6, row 3; Figures 4B,C).

**TABLE 6:** Quantitative data corresponding to Result 3.8.

Row	Cell lines	Groups	Mean ± SD	Change (%)
1	DOK	Prx1-WT	1 ± 0.02	↓88%
​	​	Prx1-WT + AZOX	0.12 ± 0	
2	Leuk1	Prx1-WT	1 ± 0.21	↓79%
​	​	Prx1-WT + AZOX	0.21 ± 0	
3	Leuk1	Prx1-WT + AZOX	0.21 ± 0	↑210%
​	​	Prx1-T90A + AZOX	0.65 ± 0.06	

### 3.9 Prx1 mutations affect energy metabolism disorder induced by AZOX in OLK cells

AZOX significantly induces mitochondrial dysfunction in OLK cells. The Trp87 and Thr90 residues of Prx1 may play important roles in mitochondrial function and bind tightly to AZOX, facilitating its pharmacological effects. To elucidate whether AZOX exerts its effect by modulating energy metabolism and whether Trp87 and Thr90 of Prx1 are involved in reprogramming energy metabolism in OLK cells, Seahorse extracellular flux assays were performed.

**FIGURE 5:**
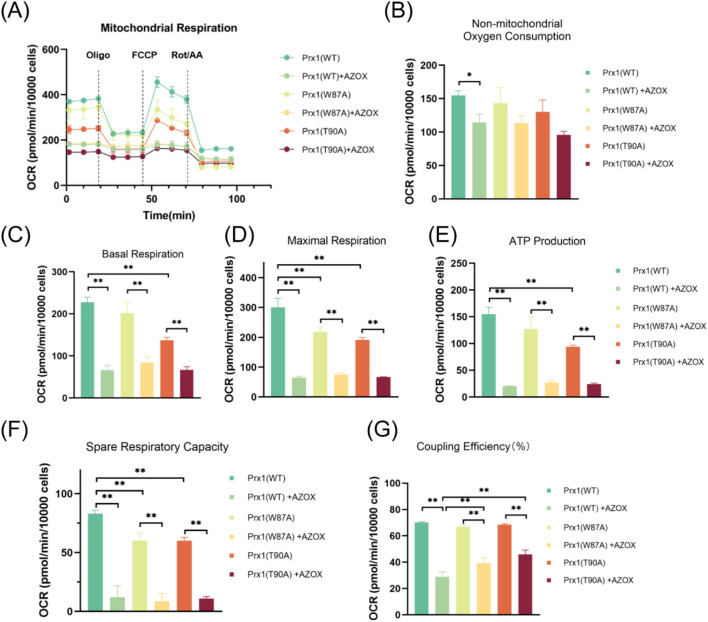
AZOX inhibits mitochondrial aerobic respiration, while Prx1 mutations attenuate the damage. **(A)** Results of the mito stress tests in Leuk1 cells are presented as real-time measurements of OCR. **(B)** Non-mitochondrial oxygen consumption. **(C)** Basal respiration. **(D)** Maximal respiration. **(E)** ATP production. **(F)** Spare respiratory capacity. **(G)** Coupling efficiency % (*P < 0.05; **P < 0.01; n = 3).

The results showed that AZOX not only inhibited mitochondrial oxygen consumption in Leuk1 cells (Figure 5A), but also suppressed non-mitochondrial oxygen consumption (Table 7, row 1; Figure 5B). AZOX significantly inhibited basal respiration (Table 7, row 2; Figure 5C), maximal respiration (Table 7, row 3; Figure 5D), ATP production (Table 7, row 4; Figure 5E), spare respiratory capacity (Table 7, row 5; Figure 5F), and coupling efficiency (Table 7, row 6; Figure 5G) in Leuk1 cells. These findings indicate that AZOX inhibits mitochondrial respiration in Leuk1 cells. Compared to Leuk1 cells transfected with wild-type Prx1, those expressing Trp87-mutated or Thr90-mutated Prx1 exhibited reduced basal respiration (Table 7, row 7; Figure 5C), maximal respiration (Table 7, rows 8-9; Figure 5D), ATP production (Table 7, row 10; Figure 5E), and spare respiratory capacity (Table 7, rows 11-12; Figure 5F), especially, more pronounced alterations were observed in Thr90-mutated Prx1 cells. Although AZOX further suppressed coupling efficiency in Prx1-mutated Leuk1 cells, the extent of inhibition was less significantly than that in wild-type Prx1-transfected cells (Table 7, rows 13-14; Figure 5G).

**TABLE 7:** Quantitative data corresponding to Result 3.9.

Row	Groups	Mean ± SD	Change (%)
1	Prx1-WT	154.92 ± 6.83	↓26%
​	Prx1-WT + AZOX	114.36 ± 12.56	
2	Prx1-WT	227.87 ± 11.87	↓71%
​	Prx1-WT + AZOX	66.12 ± 11.31	
3	Prx1-WT	300.73 ± 30.31	↓79%
​	Prx1-WT + AZOX	64.54 ± 4.93	
4	Prx1-WT	154.87 ± 13.06	↓87%
​	Prx1-WT + AZOX	20.67 ± 0.36	
5	Prx1-WT	83.16 ± 2.7	↓85%
​	Prx1-WT + AZOX	12.19 ± 9.43	
6	Prx1-WT	0.7 ± 0.01	↓59%
​	Prx1-WT + AZOX	0.29 ± 0.04	
7	Prx1-WT	227.87 ± 11.87	↓40%
​	Prx1-T90A	137.03 ± 7.19	
8	Prx1-WT	300.73 ± 30.31	↓27%
​	Prx1-W87A	218.39 ± 16.4	
9	Prx1-WT	300.73 ± 30.31	↓36%
​	Prx1-T90A	191.9 ± 7.11	
10	Prx1-WT	154.87 ± 13.06	↓39%
​	Prx1-T90A	93.88 ± 2.98	
11	Prx1-WT	83.16 ± 2.7	↓28%
​	Prx1-W87A	60.11 ± 6.31	
12	Prx1-WT	83.16 ± 2.7	↓28%
​	Prx1-T90A	60.19 ± 2.81	
13	Prx1-WT + AZOX	0.29 ± 0.04	↑34%
​	Prx1-W87A + AZOX	0.39 ± 0.04	
14	Prx1-WT + AZOX	0.29 ± 0.04	↑59%
​	Prx1-T90A + AZOX	0.46 ± 0.03	

### 3.10 AZOX is predominantly distributed in the mitochondria and nucleus of OLK cells in a targeted manner

Our previous study demonstrated that AZOX induced morphological alterations in mitochondria, extensive expansion of the endoplasmic reticulum, and an increase in the number of lipid droplets. Meanwhile, Seahorse extracellular flux assays revealed that AZOX not only inhibited the mitochondrial oxygen consumption rate but also suppressed non-mitochondrial oxygen consumption in Leuk1 cells. These results suggest that the targets of AZOX may not be restricted to the mitochondrial Qo site, but involve other intracellular targets.

**FIGURE 6:**
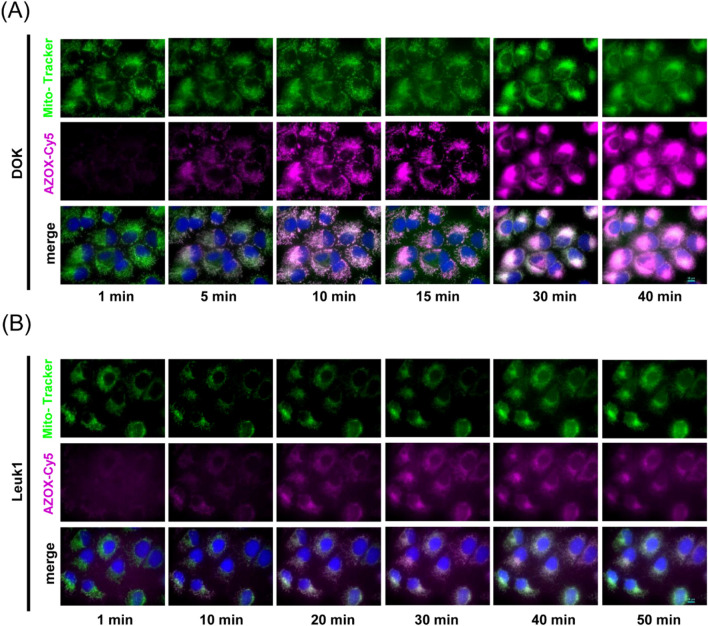
AZOX is predominantly distributed in the mitochondria and nucleus of OLK cells in a targeted manner. **(A)** Live cell imaging revealed the subcellular distribution of AZOX and mitochondrial morphological changes in DOK cells (magnification 1,000×). **(B)** Leuk1 cells (magnification 1,000×). Mitochondria (green), AZOX (pink), and nucleus (blue).

To further characterize the intracellular distribution and investigate the underlying mechanism of AZOX, we performed dynamic observation using Cy5-labeled AZOX to trace its intracellular localization and its effect on mitochondrial morphology. The results showed that fluorescent signals were detectable in DOK cells as early as 1 min after AZOX administration and gradually intensified over time, with the fluorescence mainly concentrated in the mitochondria and nucleus. Obvious mitochondrial morphological changes were observed in DOK cells at 30 min post-treatment (Figure 6A). Similar distribution patterns were observed in Leuk1 cells, and mitochondrial morphological alterations appeared at 40 min after administration (Figure 6B). These findings further confirm that AZOX can rapidly enter cells and accumulate in the mitochondria and nucleus in a targeted manner, supporting the notion that AZOX may exert its action via a multi-target, multi-site mode within cells.

### 3.11 Prx1 mutations attenuate AZOX-induced mitochondrial apoptosis in OLK cells

**FIGURE 7:**
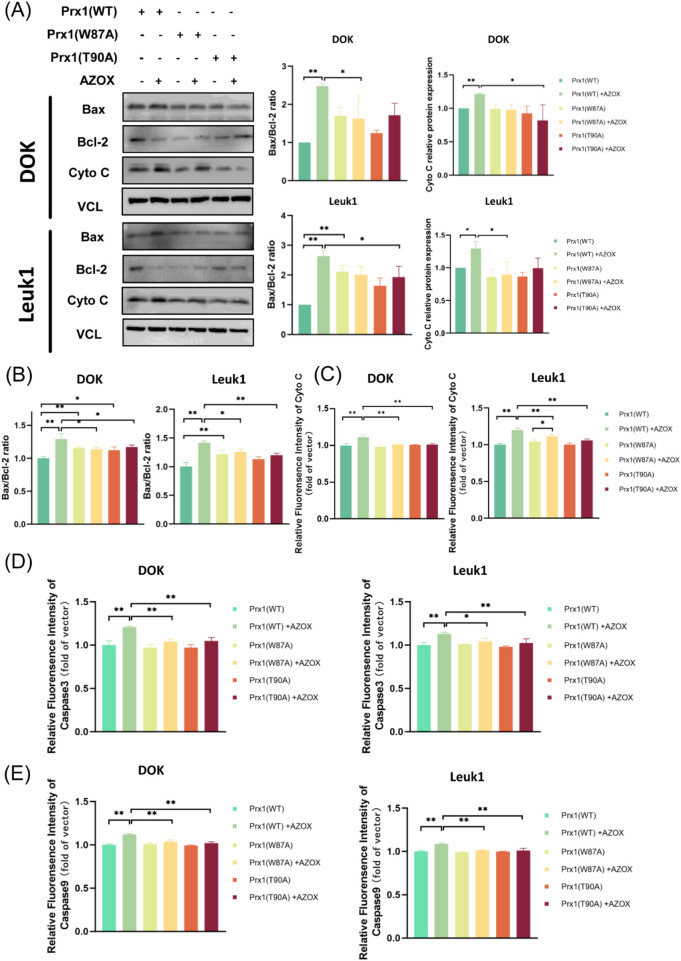
AZOX modulates the expression of mitochondrial apoptosis-related proteins while Prx1 mutations attenuate AZOX-triggered mitochondrial apoptosis in OLK cells. **(A)** Western blotting was used to detect the expression of Bax, Bcl-2 and Cyto C in OLK cells transfected with wild-type Prx1, Trp87-mutated Prx1, and Thr90-mutated Prx1. **(B–E)** The Bax/Bcl-2 ratio, Cyto C, Caspase3 and Caspase9 levels were analyzed by immunofluorescence assay (*P < 0.05; **P < 0.01; n = 3).

It is reported that apoptotic signals can stimulate the release of Cyto C from the intermembrane space of mitochondria into the cytosol. Under the regulation of BCL-2 family proteins, Cyto C binds to Apoptotic Peptidase Activating Factor 1 (APAF1) to initiate the caspase cascade, ultimately leading to apoptosis (Chipuk and Green, 2008; Czabotar et al., 2014; Zhou et al., 2024). Western blot and immunofluorescence assays demonstrated that AZOX treatment increased the Bax/Bcl-2 ratio in OLK cells transfected with wild-type Prx1 (Table 8, rows 1–2; Figures 7A,B; Supplementary Figure S1; Supplementary Figure S2). Compared to OLK cells transfected with wild-type Prx1, mutations at Trp87 in Prx1 resulted in an increased Bax/Bcl-2 ratio (Table 8, row 3). In contrast, no significant change in the Bax/Bcl-2 ratio was observed in OLK cells transfected with mutant Prx1 after AZOX treatment (Figures 7A,B; Supplementary Figure S1; Supplementary Figure S2). In DOK cells, Cyto C expression was elevated upon AZOX treatment (Table 8, row 4), whereas this effect was abolished in Prx1-mutated DOK cells (Table 8, row 5; Figures 7A,C; Supplementary Figure S3A). In Leuk1 cells, AZOX upregulated Cyto C expression (Table 8, row 6); however, the extent of upregulation was significantly attenuated in Prx1-mutated Leuk1 cells compared to wild-type transfected cells (Table 8, row 7; Figures 7A,C; Supplementary Figure S3B). Meanwhile, we found that AZOX induced elevated expression of Caspase3 (Table 8, rows 8–9) and Caspase9 (Table 8, rows 10–11) in OLK cells transfected with wild-type Prx1, whereas no significant changes in the expression levels of Caspase3 and Caspase9 were observed in OLK cells transfected with mutated Prx1 after AZOX treatment (Figures 7D,E; Supplementary Figure S4; Supplementary Figure S5). These results suggest that AZOX induces mitochondrial pathway apoptosis in OLK cells, and its efficacy is markedly reduced in OLK cells transfected with the Prx1 with Trp87 or Thr90 mutations.

**TABLE 8:** Quantitative data corresponding to Result 3.11.

Row	Cell lines	Groups	Mean ± SD	Change (%)
1	DOK	Prx1-WT	1 ± 0	↑148%
​	​	Prx1-WT + AZOX	2.48 ± 0.06	
2	Leuk1	Prx1-WT	1 ± 0	↑163%
​	​	Prx1-WT + AZOX	2.63 ± 0.21	
3	Leuk1	Prx1-WT	1 ± 0	↑111%
​	​	Prx1-W87A	2.11 ± 0.21	
4	DOK	Prx1-WT	1 ± 0	↑21%
​	​	Prx1- WT + AZOX	1.21 ± 0.01	
5	DOK	Prx1-WT + AZOX	1.21 ± 0.01	↓32%
​	​	Prx1- T90A + AZOX	0.82 ± 0.23	
6	Leuk1	Prx1-WT	1 ± 0	↑30%
​	​	Prx1- WT + AZOX	1.3 ± 0.11	
7	Leuk1	Prx1-WT + AZOX	1.3 ± 0.11	↓32%
​	​	Prx1-W87A + AZOX	0.89 ± 0.19	
8	DOK	Prx1-WT	1 ± 0.05	↑21%
​	​	Prx1- WT + AZOX	1.21 ± 0.02	
9	Leuk1	Prx1-WT	1 ± 0.03	↑13%
​	​	Prx1- WT + AZOX	1.13 ± 0.02	
10	DOK	Prx1-WT	1 ± 0.01	↑12%
​	​	Prx1- WT + AZOX	1.12 ± 0.01	
11	Leuk1	Prx1-WT	1 ± 0.01	↑9%
​	​	Prx1- WT + AZOX	1.09 ± 0.01	

## 4 Discussion

Current treatment strategies for OLK are primarily divided into surgical and non-surgical approaches (van der Waal, 2009). Surgical treatments include conventional surgical excision and laser ablation, while non-surgical options consist of photodynamic therapy and chemoprevention. Chemoprevention has become a research hotspot in OLK management. Although a variety of drugs are commonly used to treat OLK, most therapeutic outcomes remain unsatisfactory and may cause side effects such as insomnia, nervousness, abdominal pain and gastrointestinal discomfort (Palma et al., 2023). As a result, natural products have increasingly become a focus of research in the prevention and treatment of OLK.

AZOX is a novel natural strobilurin fungicide isolated from mushrooms. As an inhibitor of mitochondrial complex III, it exerts its antifungal effect by binding to the Qo site of complex III, thereby disrupting electron transport within fungal mitochondria (Esser et al., 2004). It ultimately leads to fungal death by suppressing mitochondrial respiration through the blockade of electron transfer between cytochrome b and CYC1 (Kim et al., 2007). Recently, some regulatory effect of AZOX have been discovered. It improves insulin sensitivity and reduces total fat mass in diet-induced obese mouse models through AMPK activation (Gao et al., 2014).

Our previous studies have shown that AZOX induces apoptosis in DOK cells, CAL27 and SCC15 cells, inhibits cell proliferation, and reduces the incidence of 4NQO-induced tongue oral squamous cell carcinoma (OSCC) in mice (Li et al., 2022a; Chen et al., 2020). We also found that AZOX triggers apoptosis through the PI3K/AKT and MAPK pathways, thereby suppressing the development of OLK (Li et al., 2022a). Furthermore, AZOX significantly suppresses Prx1 expression in OLK cells and targets the Prx1-CYC1 interaction to inhibit mitochondrial complex III activity, consequently impeding OLK progression (unpublished data). Moreover, AZOX exhibits a favorable biosafety profile (Chen et al., 2020). In the present study, we demonstrated that AZOX inhibited OLK cell viability and induced apoptosis by regulating the expression of mitochondrial apoptosis-related proteins. In addition, TEM showed that AZOX damaged mitochondrial structure, along with extensive endoplasmic reticulum dilation and an increase in lipid droplets. AZOX was predominantly distributed in the nucleus and mitochondria in a targeted manner as showed by live cell imaging. In addition, AZOX suppressed mitochondrial complex III activity, reduced MMP, increased mtROS level and induced oxidative stress. Furthermore, a real-time assessment of mitochondrial function using Seahorse XFe24 real-time cell metabolism analyzer showed that AZOX inhibited not only mitochondrial but also non-mitochondrial oxygen consumption in OLK cells. Additionally, AZOX significantly suppressed basal and maximal respiration, ATP production, spare respiratory capacity, and coupling efficiency. These findings indicate that AZOX compromises mitochondrial energy metabolic homeostasis in OLK cells. Our findings suggest that AZOX can inhibit mitochondrial function and energy metabolism, induce oxidative stress, and subsequently induce mitochondrial pathway apoptosis.

As a typical 2-Cys Prx enzyme, Prx1 functions not only in peroxide scavenging to alleviate oxidative stress but also, under high peroxide conditions, convert into a peroxidase-independent molecular chaperone (Kim and Jang, 2019). Prx1 is upregulated in various malignant tumors, including OSCC, and has been shown to influence tumor initiation, proliferation, apoptosis, invasion, and metastasis (Karihtala et al., 2003; Ren et al., 2013; Riddell et al., 2011). In our previous study, the analysis of The Cancer Genome Atlas (TCGA) database revealed a significant increase in Prx1 expression in head and neck squamous cell carcinoma (HNSCC), and high Prx1 expression was significantly correlated with clinical stage, tumor histological grade, and patient prognosis (Li et al., 2022b). Significantly elevated Prx1 expression was observed in OLK also, where it promoted the progression of tongue precancerous lesions (Li et al., 2024). We also found that Prx1 may be involved in AZOX inhibiting mitochondrial function (unpublished data). In the present study, it was predicted that AZOX may interact with Prx1, which suggests that Prx1 is the primary target of AZOX.

Studies have shown that covalent modifications of Prx1 can influence its peroxidase activity, hyperoxidation susceptibility, and chaperone function (Rhee and Woo, 2020). Prx1 is known to be covalently modified on multiple sites. Prx1 is hyperoxidized on Cys52; phosphorylated on Ser32, Thr90, and Tyr194; glutathionylated on Cys52, Cys83, and Cys173 (Rhee and Woo, 2020). Phosphorylation at Ser32 enhances its peroxidase activity without affecting its chaperone function (Zykova et al., 2010), whereas glutathionylation at Cys83 inhibits its chaperone activity (Fratelli et al., 2002; Fratelli et al., 2003). In our previous study, we discovered that Prx1 regulates mitophagy and inhibits cellular senescence in OLK cells via Prx1Cys52 (Lu et al., 2024). In this study, we used PyMol software to predict the binding conformation of AZOX with Prx1 and predicted their interaction sites. AZOX was found to form four hydrogen bonds with three amino acid residues of Prx1—Gln94, Thr90, and Thr49—and engage in a π-π interaction with Trp87, suggesting that Prx1 is the primary target of AZOX. Multiple studies have indicated that phosphorylation at Thr90 inactivates Prx1’s peroxidase activity and enhances its chaperone function (Lim et al., 2015; Chang et al., 2002; Jang et al., 2006). No reports have been found regarding the function of Trp87 of Prx1.

To further investigate the mechanism of AZOX on OLK, DOK and Leuk1 cells expressing Prx1 with Trp87 or Thr90 mutations were generated in this study. In OLK cells with Trp87 and Thr90 mutations in Prx1, we observed mild disruption of mitochondrial structure and compromised mitochondrial energy metabolism. Furthermore, compared to OLK cells transfected with wild-type Prx1, those carrying Trp87 or Thr90 mutations in Prx1 exhibited increased mtROS levels and decreased mitochondrial complex III activity. Most importantly, both Trp87 and Thr90 mutations reduced the efficacy of AZOX, with the Thr90 mutation having a more pronounced effect. Based on the aforementioned findings, AZOX specifically binds to the Trp87 and Thr90 sites of Prx1, thereby inducing mitochondrial dysfunction and activating the mitochondrial pathway-mediated apoptosis. It is noteworthy that mutations at the Trp87 and Thr90 sites of Prx1 can lead to conformational changes, consequently affecting its functional activity. Under high concentrations of hydrogen peroxide stress, Prx1 undergoes a molecular conformational transition, switching from its typical peroxidase function to a molecular chaperone function, thereby effectively preventing the aggregation and inactivation of other proteins and maintaining cellular homeostasis (Jang et al., 2004). In line with previous literature (Lim et al., 2015; Chang et al., 2002; Jang et al., 2006), we conclude that the Thr90 site plays an important role in maintaining the peroxidase activity of Prx1. Our results suggest that the Trp87 site may also play a critical role in the peroxidase function of Prx1, although the underlying mechanism warrants further investigation.

In the present study, employing two classical OLK cell models, DOK and Leuk1 cells, the molecular mechanism by which AZOX inhibits OLK progression via targeting the Prx1 protein was investigated, thereby providing some preclinical data for future clinical translation. To better delineate the contributions of the Trp87 and Thr90 residues within Prx1 to OLK progression and AZOX-mediated biological effects, CRISPR-Cas9 technology will be utilized in future investigations to generate Prx1 knockout cell lines and mouse models for in-depth mechanistic exploration. Nevertheless, the translation of these findings into clinical practice remains faced with multiple challenges. The current study was predominantly performed using homogeneous cell line models, whereas human OLK lesions display substantial heterogeneity at both histological and molecular levels, indicating that the generalizability of the present conclusions to patient populations warrants further validation. Moreover, the pharmacokinetic characteristics and long-term safety profiles of AZOX within the complex human tissue microenvironment remain to be systematically evaluated. Of particular note, as a mitochondrial complex III inhibitor, AZOX may exert off-target effects that could disrupt systemic energy metabolism. Meanwhile, it is reported that AZOX impairs neuronal viability, neurite outgrowth, and cortical migration in the developing brain (Kang et al., 2021), enhancing the necessity of prioritizing these safety concerns in translational research. Accordingly, future studies should not only validate the applicability of the above mechanisms in more clinically relevant complex models, but also systematically assess the effective range, potential neurotoxicity, and interactions with standard-of-care regimens through rigorous preclinical safety evaluations and dose-finding studies, thus laying a robust foundation for the initiation of subsequent clinical trials.

## 5 Conclusion

AZOX inhibits mitochondrial function and energy metabolism by binding to the Trp87 and Thr90 sites of Prx1, thereby inducing mitochondrial pathway apoptosis in OLK. The Trp87 and Thr90 sites of Prx1 play a critical role in maintaining mitochondrial homeostasis, especially with Thr90 being the key residue. AZOX could serve as a novel intervention agent for OLK.

## Data Availability

The datasets presented in this study can be found in online repositories. The names of the repository/repositories and accession number(s) can be found in the article/Supplementary Material.
