# Supplementary material for: Azoxystrobin induces mitochondrial dysfunction and mitochondrial pathway apoptosis by targeting the Prx1 Trp87 and Thr90 sites in oral leukoplakia
Source: Front Pharmacol. 2026 Apr 20;17:1769961. doi: 10.3389/fphar.2026.1769961 (PMC13135978; doi:10.3389/fphar.2026.1769961)
Supplement: Supplementary file 1 [file Supplementaryfile1.pdf]

Supplementary Figures

Supplementary Figure1

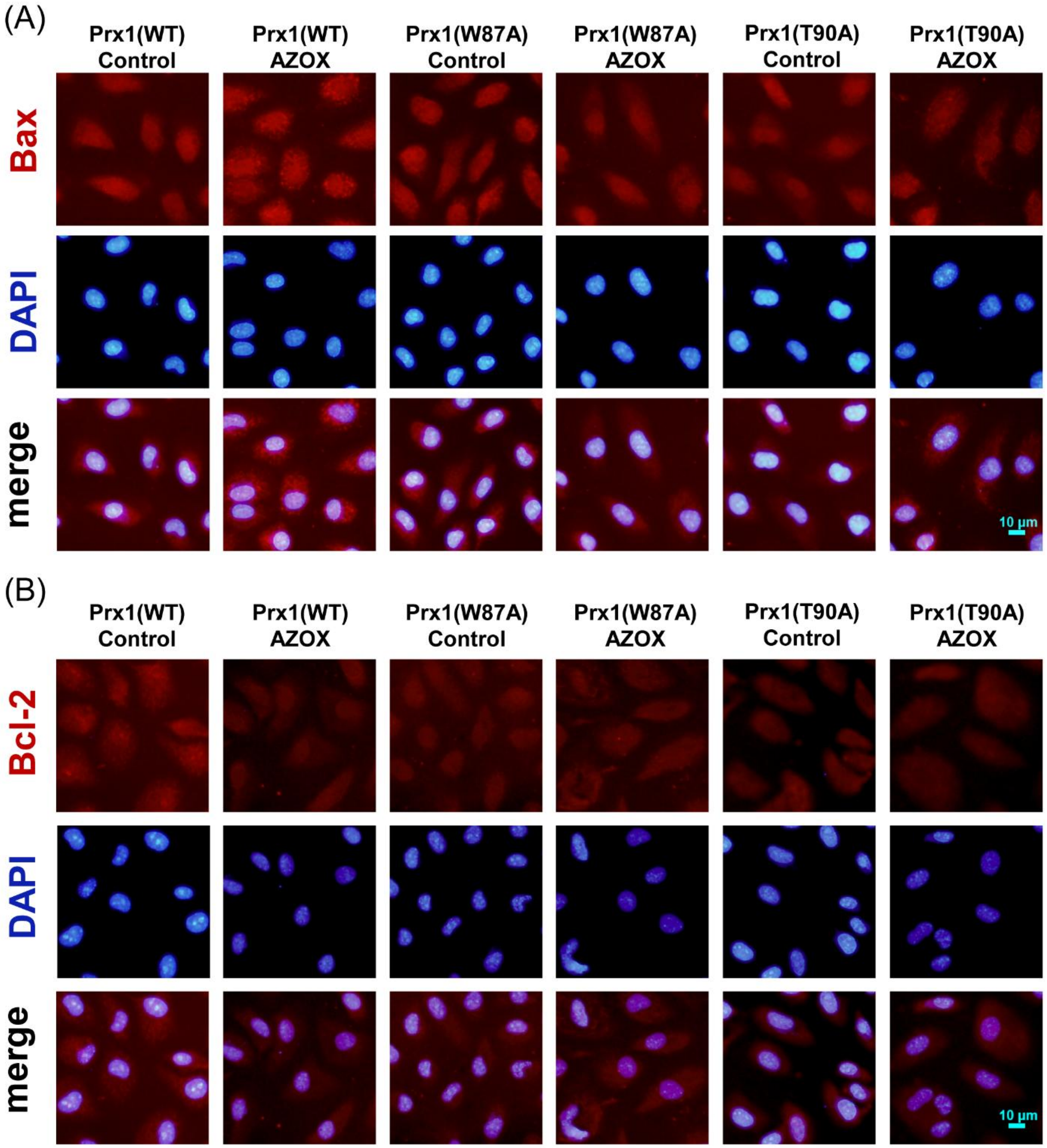

Supplementary Figure2

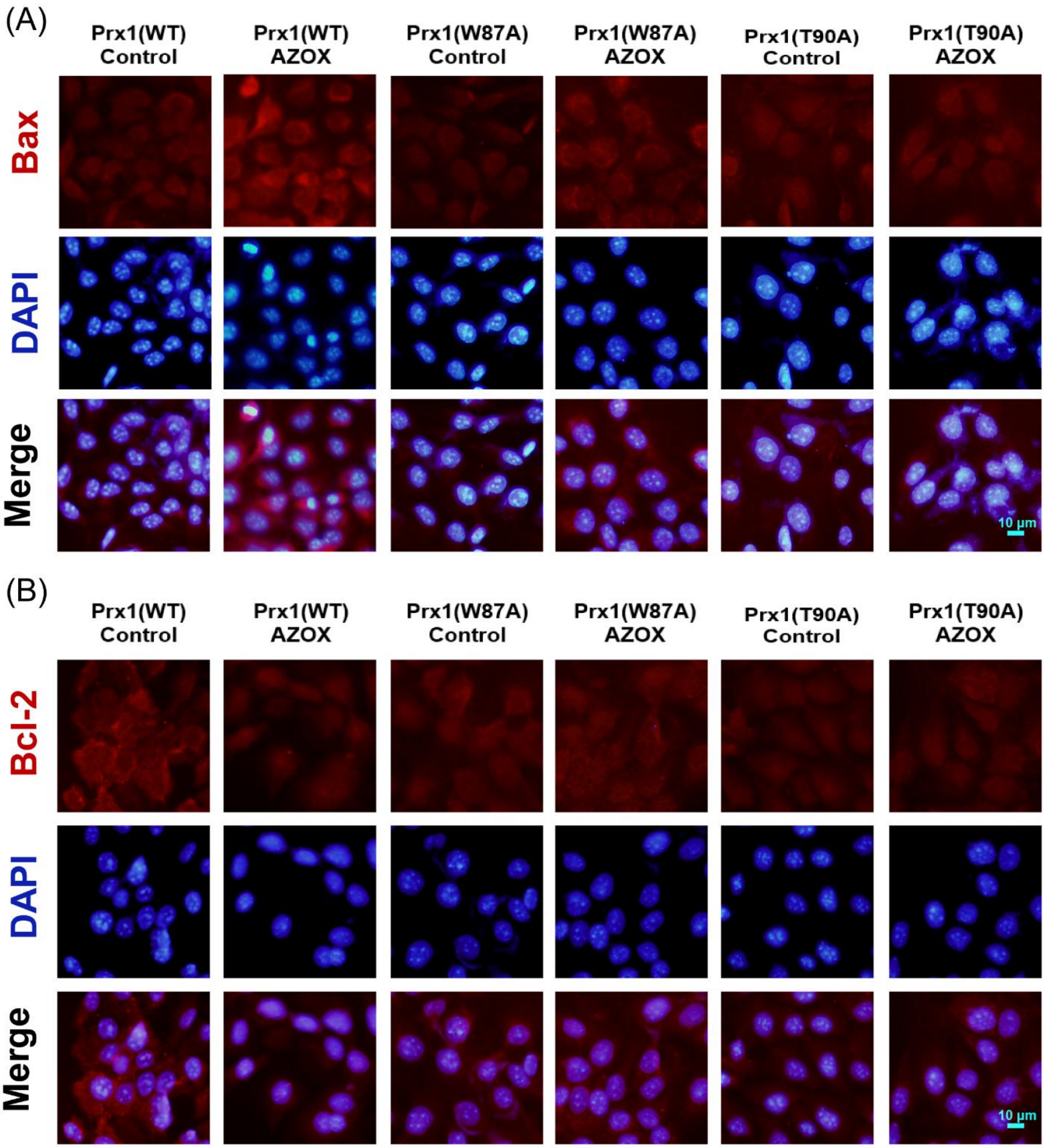

Supplementary Figure3

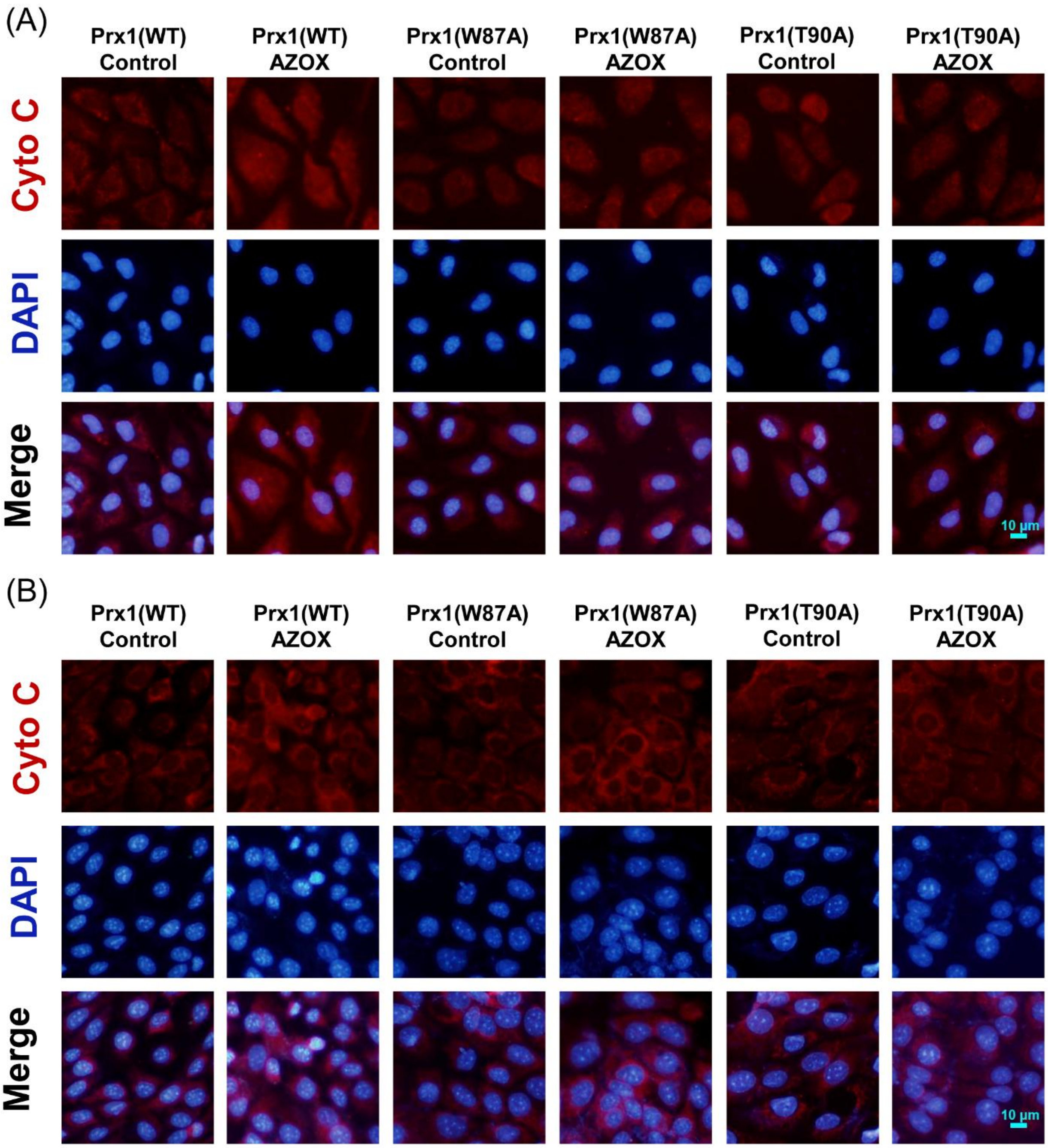

Supplementary Figure4

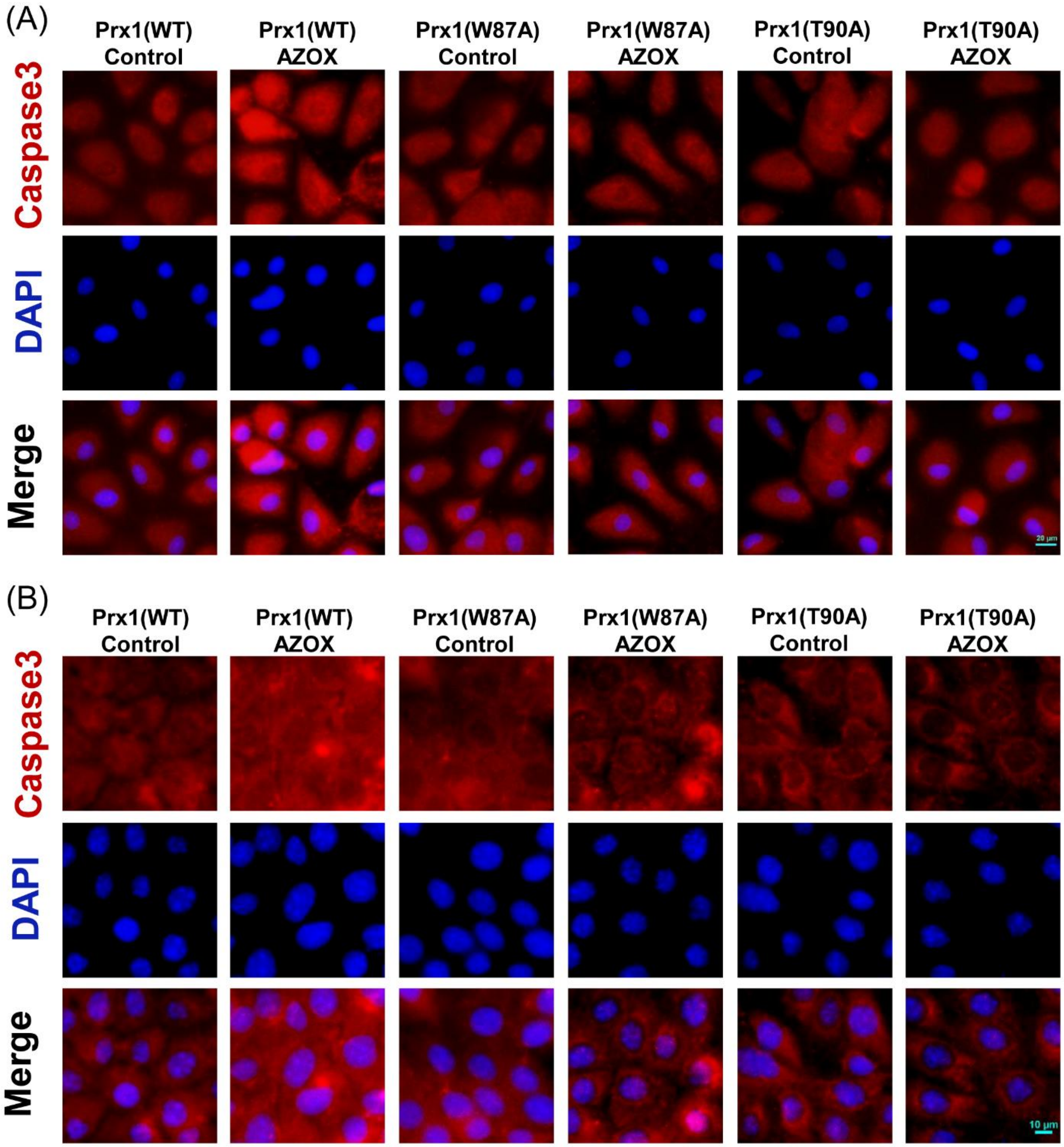

Supplementary Figure5

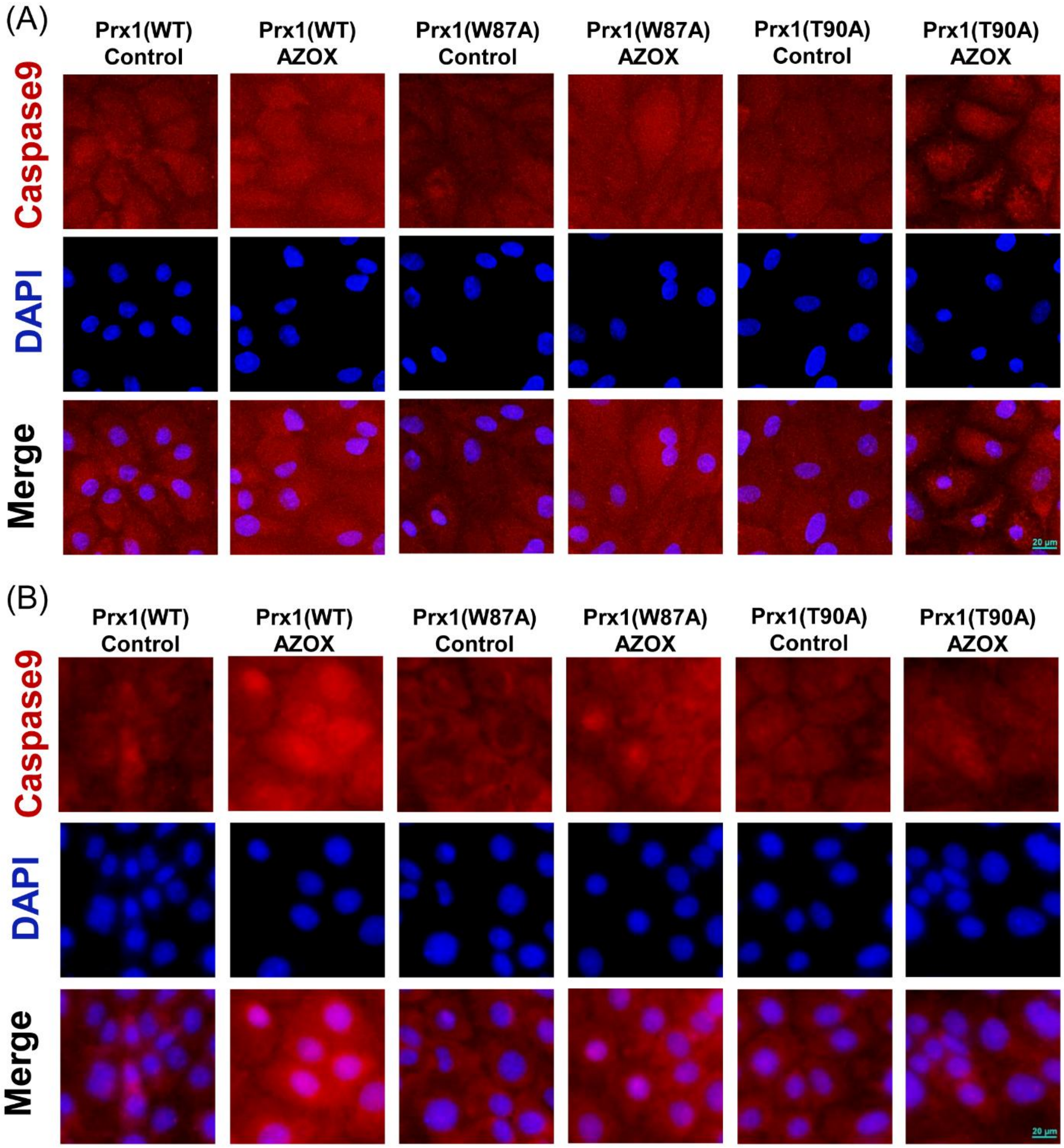

Supplementary Figure Legend

SFigure 1. AZOX elevated the Bax/Bcl-2 ratio in DOK cells, while Prx1 mutation attenuates its efficacy. **(A)** Immunofluorescence assay reflected Bax (red) distribution and DAPI (blue) in DOK cells (magnification 200×). **(B)** Immunofluorescence assay reflected Bcl-2 (red) distribution and DAPI (blue) in DOK cells (magnification 200×).

SFigure 2. AZOX elevated the Bax/Bcl-2 ratio in Leuk1 cells, while Prx1 mutation attenuates its efficacy. **(A)** Immunofluorescence assay reflected Bax (red) distribution and DAPI (blue) in Leuk1 cells (magnification 200×). **(B)** Immunofluorescence assay reflected Bcl-2 (red) distribution and DAPI (blue) in Leuk1 cells (magnification 200×).

SFigure 3. AZOX upregulates Cyto C expression in OLK cells, while Prx1 mutation attenuates this effect. **(A)** Immunofluorescence assay reflected Cyto C (red) distribution and DAPI (blue) in DOK cells (magnification 200×). **(B)** Immunofluorescence assay reflected Cyto C (red) distribution and DAPI (blue) in Leuk1 cells (magnification 200×).

SFigure 4. AZOX upregulates Caspase3 expression in OLK cells, while Prx1 mutation attenuates this effect. **(A)** Immunofluorescence assay reflected Caspase3 (red) distribution and DAPI (blue) in DOK cells (magnification 200×). **(B)** Immunofluorescence assay reflected Caspase3 (red) distribution and DAPI (blue) in Leuk1 cells (magnification 200×).

SFigure 5. AZOX upregulates Caspase9 expression in OLK cells, while Prx1 mutation attenuates this effect. **(A)** Immunofluorescence assay reflected Caspase9 (red) distribution and DAPI (blue) in DOK cells (magnification 200×). **(B)** Immunofluorescence assay reflected Caspase9 (red) distribution and DAPI (blue) in Leuk1 cells (magnification 200×).
